# Metabolomics in Acute Kidney Injury: The Clinical Perspective

**DOI:** 10.3390/jcm12124083

**Published:** 2023-06-16

**Authors:** Daniel Patschan, Susann Patschan, Igor Matyukhin, Oliver Ritter, Werner Dammermann

**Affiliations:** 1 Department of Medicine 1, Cardiology, Angiology, Nephrology, Brandenburg Medical School Theodor Fontane, University Hospital Brandenburg, 14770 Brandenburg, Germany; 2 Faculty of Health Sciences Brandenburg, Brandenburg Medical School Theodor Fontane, 15562 Rüdersdorf bei Berlin, Germany; 3 Department of Medicine 2, Gastroenterology, Diabetes, Endocrinology, Brandenburg Medical School Theodor Fontane, University Hospital Brandenburg, 14770 Brandenburg, Germany

**Keywords:** AKI, metabolomics, KRT, recovery of kidney function, survival

## Abstract

Background: Acute kidney injury (AKI) affects increasing numbers of hospitalized patients worldwide. The diagnosis of AKI is made too late in most individuals since it is still based on dynamic changes in serum creatinine. In recent years, new AKI biomarkers have been identified; however, none of these can reliably replace serum creatinine yet. Metabolomic profiling (metabolomics) allows the concomitant detection and quantification of large numbers of metabolites from biological specimens. The current article aims to summarize clinical studies on metabolomics in AKI diagnosis and risk prediction. Methods: The following databases were searched for references: PubMed, Web of Science, Cochrane Library, and Scopus, and the period lasted from 1940 until 2022. The following terms were utilized: ‘AKI’ OR ‘Acute Kidney Injury’ OR ‘Acute Renal Failure’ AND ‘metabolomics’ OR ‘metabolic profiling’ OR ‘omics’ AND ‘risk’ OR ‘death’ OR ‘survival’ OR ‘dialysis’ OR ‘KRT’ OR ‘kidney replacement therapy’ OR ‘RRT’ OR ‘renal replacement therapy’ OR ‘recovery of kidney function’ OR ‘renal recovery’ OR ‘kidney recovery’ OR ‘outcome’. Studies on AKI risk prediction were only selected if metabolomic profiling allowed differentiation between subjects that fulfilled a risk category (death or KRT or recovery of kidney function) and those who did not. Experimental (animal-based) studies were not included. Results: In total, eight studies were identified. Six studies were related to the diagnosis of AKI; two studies were performed on metabolic analysis in AKI risk (death) prediction. Metabolomics studies in AKI already helped to identify new biomarkers for AKI diagnosis. The data on metabolomics for AKI risk prediction (death, KRT, recovery of kidney function), however, are very limited. Conclusions: Both the heterogenous etiology and the high degree of pathogenetic complexity of AKI most likely require integrated approaches such as metabolomics and/or additional types of ‘-omics’ studies to improve clinical outcomes in AKI.

## 1. Introduction

Acute kidney injury (AKI) evolves in increasing numbers of hospitalized patients worldwide. The overall in-hospital incidence has been shown to range from 15 to 30% [1], and the average mortality of all individuals with nosocomial AKI varies between 15 and 20%. Under intensive care conditions, however, more than 60% of all treated subjects are affected by acute kidney excretory dysfunction [2], and the mortality exceeds 50%, even if kidney replacement therapy has been initiated [2]. The kidney function of surviving patients may recover completely, incompletely, or not at all [3]. The criteria of kidney recovery are heterogenous; an ultimate definition is still missing [4]. Nevertheless, every individual AKI episode increases the risk for chronic kidney disease (CKD) in the years to come [5,6]. The transition from AKI to CKD includes AKD (acute kidney disease), the term describes persistent excretory dysfunction or failure from day eight until the end of month three [7]. Finally, post-AKI survivors are at a higher lifetime risk of death in general [8]. One reason for the poor prognosis is certainly the inability to make the diagnosis of AKI in time. Since 2012, AKI has been diagnosed according to the ‘KDIGO clinical practice guidelines for acute kidney injury’ [9]. The criteria are based on dynamic changes in serum creatinine, despite substantial limitations of the parameter. Hence, clinical researchers have strived to identify alternative AKI biomarker molecules for many years now. Meanwhile, three groups of AKI biomarkers have been defined: stress, damage, and functional markers [10]. Most likely, future AKI definitions will incorporate selected members of the damage marker group [11]. Nevertheless, no individual biomarker or combination of biomarker molecules can truly replace serum creatinine so far.

The principal aim of the so-called ‘-omics’-concept is to elucidate cellular/tissue response patterns under both physiological and pathological conditions in a more integrated fashion. The term ‘-omics’ represents datasets on the detection, quantification, and characterization of biological molecules. The following types of ‘-omics’ have been proposed as the ‘big four’: genomics, transcriptomics, proteomics, and metabolomics [12]. Particularly metabolomics offers new perspectives in AKI biomarker research; it encompasses the analysis of lower molecular weight (<1.5 kD) substances in cells/certain types of tissue/biological fluids. Herein, we aim to review the literature on metabolic profiling in AKI diagnosis and risk prediction.

## 2. Methods

**Figure 1:**
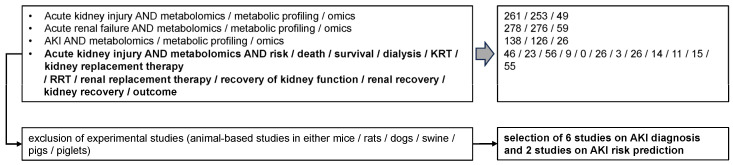
Search strategy for the identification of articles included in the study.

The following databases were searched for references: PubMed, Web of Science, Cochrane Library, and Scopus, and the period lasted from 1940 until 2022. The following terms were utilized: ‘AKI’ OR ‘Acute Kidney Injury’ OR ‘Acute Renal Failure’ AND ‘metabolomics’ OR ‘metabolic profiling’ OR ‘omics’ AND ‘risk’ OR ‘death’ OR ‘survival’ OR ‘dialysis’ OR ‘KRT’ OR ‘kidney replacement therapy’ OR ‘RRT’ OR ‘renal replacement therapy’ OR ‘recovery of kidney function’ OR ‘renal recovery’ OR ‘kidney recovery’ OR ‘outcome’. Studies on AKI risk prediction were only selected if metabolomic profiling allowed differentiation between subjects that fulfilled a risk category (death or KRT or recovery of kidney function) and those who did not. Experimental (animal-based) studies were not included. Figure 1 summarizes the search strategy.

## 3. Metabolic Profiling

### 3.1. Definition

Metabolic profiling, also referred to as metabolomics, is the analysis of lower molecular weight (<1.5 kD) metabolites in biological systems/specimens. The latter potentially encompasses individual cells, certain types of tissue, body fluids, or even nutritional components. The term ‘-omics’ represents datasets on the detection, quantification, and characterization of biological molecules [13]. Dettmer et al. [14] summarized the so-called ‘-omics-cascade’ in 2007; it consists of four elements: genomics, transcriptomics, proteomics, and metabolomics. Under ideal conditions, the integration of data from every element shall increase the understanding of biological responses to diseases and/or environmental factors. In addition to the ‘big four’ [12], other omics have also been defined, for instance, epiomics (epigenetic/epitranscriptomic/epiproteomic modifications) and interactomics (DNA–protein, DNA–RNA, RNA–RNA, and RNA–protein interactions).

### 3.2. Methodical Considerations

The first reference to metabolic profiling in a broader sense was published in 1948. Williams and Kirby [15] performed paper chromatography for the analysis of urine and saliva from schizophrenic subjects. In 2022, Dai et al. [12] extensively reviewed methodical approaches that have been employed for -omics analyses since then. The two principal procedures in use are sequencing and mass spectrometry (MS). While the former is indispensable in all types of studies on nucleic acids, the latter is being applied in prote- and metabolomics. Various spectrometrical techniques are available, all associated with certain advantages and disadvantages: FT-IR (Fourier-transform infrared) [16,17], Raman [18], and NMR (nuclear magnetic resonance [19]) spectroscopy, and different types of mass spectrometry (e.g., LC-MS—liquid chromatography coupled to mass spectrometry [20]). ‘Spectroscopy’ per se is the ‘science of studying the interaction between matter and radiant energy’. It should, in theory, not be employed for describing distinct technical procedures. Apart from the specific method used, metabolic profiling may either be performed as non-targeted or targeted metabolomics [12,21]. While the former is intended to identify new candidates of interest to generate hypotheses, the goal of the latter is to confirm a specific hypothesis through the analysis of certain pre-defined metabolites.

In the following paragraphs, the methods used and the general approach (non-targeted vs. targeted) for metabolic profiling will be mentioned if necessary.

## 4. Acute Kidney Injury

### 4.1. Definition and Epidemiology

Acute kidney injury (AKI) must be diagnosed if one of the following criteria is (are) fulfilled: an increase in serum creatinine of at least 0.3 mg/dL or a 1.5-fold increase within seven days or a reduction in urine output to under 0.5 mL/kg/h for 6 h or longer [9]. Three KDIGO stages of AKI must be distinguished, depending on the relative increase in serum creatinine as compared to the baseline ((1): 1.5–1.9 baseline; (2): 2.0–2.9 baselines; (3): 3 times baseline or higher). Stage 3 must also be diagnosed if KRT (kidney replacement therapy) is mandatory or if the absolute serum creatinine surpasses 353.6 micromol/L, or if the eGFR (estimated glomerular filtration rate) falls below 35 mL/min/1.73 m^2^. The last criterion exclusively applies to patients younger than 18 years. Finally, urine output over time may also indicate the stage, independently from dynamic changes in serum creatinine [9]. AKI evolves in increasing numbers of in-hospital patients. Hoste and colleagues reported nosocomial incidences of 18–30% [1]. Under intensive care conditions, however, 30–60% of all treated subjects are variably affected by an acute decline in kidney excretory function. ICU (intensive care unit) patients with KRT (kidney replacement therapy)-requiring AKI are at an overall risk of death of 50% [22]. A comparably poor prognosis has lately been reported in cardiorenal syndrome type 3 [23]. The coincidence of hematological neoplasia, chemotherapy-associated sepsis, and KRT-requiring AKI has been associated with 100% mortality [24]. In the long term, every individual AKI episode increases the risk for chronic kidney disease (CKD) [5,6]. Finally, AKI also shortens the overall life expectancy. Rewa and colleagues [8] reported a stepwise decrease in long-term survival with increasing AKI severity according to RIFLE [25].

### 4.2. Biomarkers in AKI Diagnosis and Risk Prediction

Serum creatinine is far from being a so-called optimal biomarker. Kidney excretory function must decline by at least 60% until serum creatinine rises. The marker is by no means specific and offers almost no prognostic value at all. In addition, creatinine does not reflect structural kidney damage that is not associated with diminished excretory function. This particular condition has been defined as ‘subclinical AKI’ [26]. Therefore, alternative AKI biomarkers have been intensively studied for more than 20 years. In general, three groups of biomarkers must be distinguished: markers of stress, structural damage, or excretory (dys)function [27]. Currently, the third group is exclusively represented by cystatin C [28] and proenkephalin A [29]. The stress marker group encompasses dickkopf-3 [30] and the product of tissue metalloproteinase-2 and insulin-like growth factor binding protein-7 [31]. The two latter constitute the Nephrocheck^®^ test [32], a commercially available kit for early AKI recognition. The majority of all biomarkers identified so far belong to the damage marker group. The literature offers countless studies on the role of damage biomarkers in various types of AKI [33]. Thus, the ‘Acute Disease Quality Initiative Consensus Conference’ on AKI biomarkers was held in 2019; a summarizing report was published in 2020 [10]. The clinical usability of individual biomarkers was evaluated in relation to five outcome categories: risk assessment, AKI prediction, AKI diagnosis, severity, and kidney recovery. One additional, quite important category was, however, not considered, the prediction of in-hospital death. Only a few studies assessed the role of biomarker-based mortality prediction in AKI in general [20,34,35,36]. One study, nevertheless, employed a metabolomics-based approach [20]. In the future, selected damage biomarkers will most likely be incorporated in AKI definition criteria [11]; an updated version of the ‘KDIGO clinical practice guidelines for acute kidney injury’ [9] is, however, still pending.

The following paragraphs will summarize all relevant clinical studies on metabolomics regarding AKI diagnosis and risk prediction. The risk categories of interest are KRT, in-hospital death, and recovery of kidney function. Experimental studies will not be discussed since the topic has been addressed in a separate article.

### 4.3. Metabolomics in AKI Diagnosis

A 2016 study published by Elmariah et al. [37] was performed on patients that received transcatheter aortic valve replacement (TAVR). The diagnosis of AKI was made according to the 2012 published ‘Valve Academic Research Consortium-2 criteria’ [38], which correspond to the AKIN criteria [25]. In total, 44 patients were included, of whom 22 had the diagnosis of CKD (chronic kidney disease [39]). Nine individuals acquired TAVR-associated AKI. Metabolic profiling was performed with liquidchromatography–mass spectrometry; comparisons were made with 2164 individuals from the Framingham Heart Study [40]. In patients with pre-existing CKD, numerous serum metabolites were detected differentially (total number of analytes: 85—selection: 5-adenosylhomocysteine, Xanthosine, Cysteamine, Kynurenic acid, Taurine, Betaine, Carnitine, Phenylalanine, Citrulline, Homocysteine, Thymidine, and others). Six metabolites correlated with the baseline eGFR (estimated glomerular filtration rate) negatively. Finally, one metabolite, 5-adenosylhomocysteine, was significantly associated with dynamic changes in serum creatinine. It predicted AKI even after the adjustment for baseline eGFR. The AKI probability increased with increasing tertile of baseline 5-adenosylhomocysteine: 50% of the subjects in the highest tertile developed AKI as opposed to none in the lowest.

In 2018, Zhang et al. [41] performed a single-center study on kidney transplant recipients. All allografts were explanted from living donors. The total patient number was 42, of whom 30 had developed AKI. The control group consisted of 24 healthy subjects. Metabolic analyses were conducted with ultra-high performance liquid chromatography-tandem mass spectrometry (UHPLC–MS/MS). The study targeted the amino acid (AA) metabolism (selected metabolites: alanine, glycine, leucine, proline, serine, threonine, valine, and others). Particularly the metabolism of two amino acids was affected, respectively: tryptophan and arginine. Firstly, serum concentrations of both amino acids were lower in transplant recipients than in controls. Comparable differences were shown for other AAs also. Next, tryptophan was lower in AKI individuals than in transplant patients without acute kidney injury. Finally, prediction analysis revealed an AUC of 0.9 regarding the diagnosis of AKI if symmetric dimethylarginine (SDMA) and tryptophan were combined. Exact information about the time frame during which blood samples were collected was not provided by the authors; it remains uncertain whether blood was drawn before, in parallel, or after AKI onset.

A Chinese study from 2021 [42] favored a non-targeted approach using ‘ultra-high-performance liquid chromatography-tandem quadrupole time-of-flight mass spectrometry’ (UPLC-Q/TOF–MS). Blood and urine samples were collected from 30 AKI subjects and 20 healthy controls; both groups were comparable in age and gender distribution. Metabolic urine profiling revealed differences between AKI and controls in the following substances: 2-S-glutathionyl glutathione acetate, 5-l-Glutamyl-taurine, and l-Phosphoarginine (other metabolites were not exclusively listed but termed by numbers—factor 10 to factor 2940). All three components were positively correlated with serum creatinine. The metabolites were shown to be involved in the following processes: cytochrome P450, arginine, and proline metabolism. Potential pathophysiological implications were speculative; however, the three molecules were proposed as possible diagnostic biomarkers for AKI diagnosis.

An also more recent study (2021—[43]) investigated samples from patients with vancomycin-associated AKI (*n* = 28). Additional groups were (I) patients that received vancomycin for infection control but who did not acquire AKI (*n* = 23), (II) chronic kidney disease subjects (*n* = 23), and 23 healthy persons. No individual from any group required KRT. The method used was ‘Triple Quadrupole MS/MS’ [44]. Although not defined by the authors, the study targeted serotonin (5-hydroxytryptamin—5-HT) and the serotonin metabolite 5-hydroxyindoleacetic acid (5-HIAA). Both molecules are involved in experimental ischemia/reperfusion damage [45]. Oxidative stress, on the other hand, has been proposed as a key mechanism of vancomycin-associated kidney damage [46]. Metabolic profiling was not restricted to these two substances; in total, 43 amino acids and respective derivates underwent profiling. Numerous metabolites differed between patients with vancomycin-associated AKI and all other individuals. For instance, beta-alanine, anserine, gammaaminobutyric acid, aspartic acid, ethanolamine, glutamic acid were lower in the AKI group. Higher serum levels were shown for beta-aminoisobutyric acid, citrulline, 3-methylhistidine (and others). Comparisons between AKI subjects and all other groups revealed the following differences: 5-HT was lower, and 5-HIAA and the 5-HIAA/5-HT ratio were both higher in patients with acute kidney injury. AUC-ROC analyses showed the ratio to be AKI predictive compared to all other groups and every individual subgroup with AUC values ranging between 0.79 and 0.93. The 5-HIAA/5-HT ratio was finally proposed as a potential surrogate marker of vancomycin-associated AKI.

Tian et al. [47] performed a study on patients that received (on-pump) coronary artery bypass surgery. Since AKI frequently evolves in post-cardiac surgery subjects, the authors aimed to identify metabolic panels that predict acute kidney injury during follow-up. Metabolomic studies were performed in urine samples, and a total number of 159 patients was included. Fifty-five patients acquired AKI post-surgery; in 104 individuals, kidney excretory function remained unaffected. Finally, the concentrations of 28 distinct substances differed between AKI and non-AKI patients. A combination of five metabolites (tyrosyl-gamma-glutamate, deoxycholic acid glycine conjugate, 5-acetylamino-6-amino-3-methyluracil, arginyl-arginine, and L-methionine) was shown as a powerful AKI predictor with an area under the curve of 0.89.

Another urine-based study on AKI prediction was published in 2022 [48]. Urine samples were collected from children, with the following group distribution: pre-AKI (*n* = 15), established AKI (*n* = 22), and controls (*n* = 30). Patients were recruited from the respective cohorts of two older studies, from which the first compared cystatin C with serum creatinine in critically ill children [49]. The second trial was also performed in pediatric ICU patients; it aimed to compare certain biomarkers for early AKI diagnosis and grading [50]. The initial urine samples were collected within a maximum of three days after ICU admission; afterwards, samples were stored daily until days 5 or 14. Almost 200 metabolites were detected/quantified, including water-soluble and insoluble substances. The study revealed 20 metabolic candidates (acetylornithine, serotonin, C5.DC..C6.OH., aspartic acid, arginine, methionine, methylmalonic acid, C2, histidine, taurine, C7.DC, homovanillic acid, PC.aa.C34.1, glutamine, kynurenine, PC.aa.C34.1, SM.C16.0, C9, C4, C3.DC..C4.OH) that contributed to a so-called discriminant score (dscore) regarding pre-AKI. Some metabolites were included in both dscores (pre-AKI and AKI). Further marker elimination revealed 13 metabolites as AKI predictive up to three days before AKI onset (AUC above 0.9). The respective metabolic profile showed similarities with profile characteristics at the time of AKI onset. The overlap between the two profiles indicated metabolic patterns of acute kidney damage that appear prior the clinical diagnosis of AKI, which is still based on dynamic changes in serum creatinine. The latter aspect may not be underestimated since an earlier AKI diagnosis allows earlier therapeutic interventions.

In summary, recent studies on metabolic profiling in AKI revealed several new molecules that differ between affected and non-affected individuals, either in serum or urine. Some metabolites, such as the combination of symmetric dimethylarginine (SDMA) and tryptophan [41] or the 5-HIAA/5-HT ratio [43], have even been identified as AKI predictive with an AUC of 0.9 or above.

### 4.4. Metabolomics in AKI Risk Prediction

The data on metabolomics for AKI risk prediction are limited. As mentioned earlier, the risk categories of interest are in-hospital death, the transient or persistent need for KRT, and recovery of kidney function. As summarized by Ostermann et al. [10], at least three biomarkers have been shown to offer information on the chance of recovery post-AKI (C-C motif chemokine ligand 14 [51], hepatocyte growth factor [52], and proenkephalin A [29]). In 2021, Gisewhite et al. [53] published a secondary analysis of urine samples from 82 combat casualties who were earlier investigated by Stewart and colleagues (‘The potential utility of urinary biomarkers for risk prediction in combat casualties: a prospective observational cohort study’ [54]). Study inclusion was only possible if the injury had occurred not later than 48 h before admission. Technically, the authors used proton nuclear magnetic resonance (1H-NMR) spectroscopy. Nine out of 73 analysed urine metabolites were associated with death and the need of KRT during follow-up (lactate, glucose, 1-methylnicotinamide, 2-hydroxybutyrate, glycine, pyruvate, 2-hydroxyvalerate, 1,6-anhydro-beta-D-glucose, threonine), 11 parameters were associated with the AKI stage (1-methylnicotinamide, lactate, glycine, citrate, 3-hydroxyisovalerate, hippurate, histidine, xanthosine, 3-indoxylsulfate, tartrate, threonine, phenylacetylglycine, 1,6-anhydro-beta-D-glucose, glucose, pyruvate, indole-3-acetate). Finally, two metabolites were identified as outcome predictors: increased 1-methylnicotinamide was associated with mortality, KRT, and higher AKI stages, an increase of glycine, in contrast, was indicative of survival, no need for KRT, and less severe acute kidney injury.

Sun and colleagues [20] performed an observational trial designed as a subgroup analysis of the so-called ATN study [55]. The latter compared intensive with less intensive KRT in AKI subjects at the ICU. Sun et al. [20] employed 202 individual blood samples from a total number of 1124 patients that had been included in the ATN trial. Two groups were defined: day one patients (blood was collected on day one after study inclusion—*n* = 97) and day eight patients (blood was collected on day eight after study inclusion—*n* = 105). Survival numbers in the two groups were *n* = 46 on day eight (day one patients) and *n* = 80 on day 28 (day eight patients). The authors revealed significant differences in metabolite concentrations between survivors and non-survivors, respectively. In the end, several mortality-predictive metabolite combinations were identified. For instance, four distinct markers (1-arachidonoyl-lysoPC, 1-eicosatetraenoyl-IysoPC, 5-methylthioadenosine, tyrosine) were death-predictive regarding day eight (AUC 0.64), whereas a panel of 11 metabolites (3-Dehydroxycarnitine, 2,3-diaminopropionate, citrulline, creatine, hippurate, LysoPC(16:0), LysoPC(16:1), LysoPC(18:2), 1-arachidonoyl-lysoPC, 1-eicosatetraenoyl-IysoPC, tyrosine) was death predictive regarding day 28 (AUC 0.78).

Table 1 summarizes all selected studies, including reference/year of publication, design, and outcomes, respectively.

**Figure 2:**
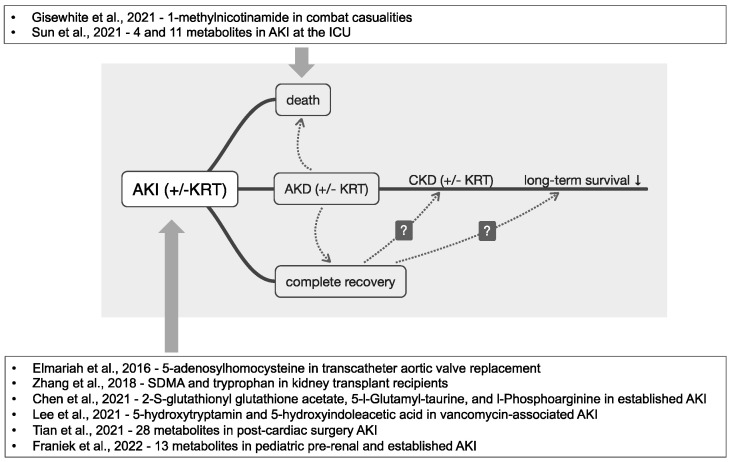
Clinical courses of AKI and references on metabolic profiling in AKI diagnosis and risk prediction. AKI is associated with an increased risk of in-hospital death; in surviving individuals, kidney function, however, may either recover completely, incompletely, or not at all. The transition to CKD includes AKD in some patients. The general life expectancy is reduced in all individuals that suffered from AKI at least once. Currently, metabolic profiling has been performed related to the diagnosis of AKI, and two studies employed metabolomics for AKI risk prediction (abbreviations: AKI—acute kidney injury, AKD—acute kidney disease, CKD—chronic kidney disease, KRT—kidney replacement therapy) [20,37,41,42,43,47,48,53].

Figure 2 summarizes the clinical course of AKI patients and all cited references on metabolomics studies in AKI diagnosis and risk prediction.

## 5. Conclusions

Metabolomics studies in AKI already helped to identify new biomarkers for AKI diagnosis.

One study even revealed AKI-related metabolic patterns that emerged several days before the clinical diagnosis of the syndrome.

The data on metabolomics for AKI risk prediction (death, KRT, recovery of kidney function), however, are very limited.

Both the heterogenous etiology and the high degree of pathogenetic complexity of AKI most likely require integrated approaches such as metabolomics and/or additional types of omics studies to improve clinical outcomes in AKI.

## Figures and Tables

**Table 1:** Summary of all selected studies on the role of metabolomics in AKI diagnosis and risk prediction.

Reference/Year	Design	Findings
AKI diagnosis		
Elmariah et al., 2016 [37]	prospective, observational; transcatheter aortic valve replacement (TAVR) patients (*n* = 44) and participants of the Framingham Heart Study (*n* = 2164)	5-adenosylhomocysteine AKI predictive, even after adjustment for baseline creatinine
Zhang et al., 2018 [41]	kidney transplant recipients, living donors; *n* = 42 (AKI in *n* = 30), 24 healthy subjects as controls	tryptophan lower in transplant patients than in controls and lower in AKI than in non-AKI patients; AKI prediction through tryptophan and symmetric dimethylarginine (SDMA) in combination (AUC 0.9)
Chen et al., 2021 [42]	30 AKI subjects and 20 healthy controls, gender- and age-matched	glutathione acetate, 5-l-Glutamyl-taurine, and l-Phosphoarginine higher in AKI, positive correlation with serum creatinine
Lee et al., 2021 [43]	four groups: vancomycin-associated AKI, vancomycin treatment without AKI, CKD, and healthy controls	identification of the 5-hydroxyindoleacetic acid/5-hydroxytryptamin-ratio as surrogate marker of vancomycin-associated AKI
Tian et al., 2021 [47]	patients that receive (on-pump) coronary artery bypass surgery; post-surgery AKI in *n* = 55, stable kidney function in *n* = 104	identification of 5 AKI predictive urine metabolites
Franiek et al., 2022 [48]	urine samples from children with either pre-AKI (*n* = 15), established AKI (*n* = 22), and controls (*n* = 30)	20 metabolites discriminated between pre-AKI and established AKI, 13 metabolites predicted AKI up to 3 days in advance
AKI risk prediction		
Gisewhite et al., 2021 [53]	urine analysis from 82 individuals with combat injury, injury occurred not later than 48 h before inclusion	increased 1-methylnicotinamide was associated with mortality, KRT, and higher AKI stages
Sun et al., 2021 [20]	202 AKI individuals from the ATN study cohort [55]	4/11 serum metabolites death predictive for days 8/28

## Data Availability

Not applicable.
